# Ramadan fasting practices and acute complications among Arab children, adolescents, and young adults with diabetes: a cross-sectional survey

**DOI:** 10.3389/fendo.2026.1867277

**Published:** 2026-08-24

**Authors:** Aqeel Alaqeel

**Affiliations:** Department of Pediatrics, College of Medicine, Qassim University, Buraidah, Saudi Arabia

**Keywords:** adolescents, Arab countries, children, counseling, fasting, Ramadan, type 1 diabetes, type 2 diabetes

## Abstract

**Introduction:**

Fasting during Ramadan poses specific challenges for children and adolescents with diabetes and may adversely affect glycemic control and the risk of acute complications. Data on the safety of Ramadan fasting among Arab youth with diabetes and adherence to guideline-based care remain limited. This study aimed to evaluate the experiences of Ramadan fasting among Arab children, adolescents, and youths with diabetes.

**Methods:**

In this cross-sectional study, we used an expert-reviewed and pilot-tested questionnaire distributed through social media to recruit a non-probabilistic online convenience sample of children, adolescents, and young adults with diabetes across Arab countries. All demographic, clinical, counseling, and fasting-related outcomes were self-reported.

**Results:**

In total, 242 (53.7% female) patients participated in this study. The most common age group was 13–17 years (50.8%), and 95.9% of the patients had type 1 diabetes. Before Ramadan, 11.5% reported diabetic ketoacidosis (DKA), 5.0% reported severe hypoglycemia during the previous 3 months, and 26.0% had hypoglycemia unawareness. Nearly half (45.5%) of the participants reported being permitted to fast by their physicians. During Ramadan, DKA and severe hypoglycemia occurred in 6.2% and 2.5% of the patients, respectively. The median number of completed fasting days was 22 (range 0–30). Older patients (aged 13–25 years) were more likely to be allowed to fast (p = 0.033) and complete more fasting days (p < 0.001). In multivariate regression, older age independently predicted fasting allowance (AOR = 1.951, 95% CI: 1.132 – 3.363, p = 0.016). Multiple daily injections decreased the likelihood of being allowed to fast (AOR = 0.444, 95% CI: 0.230 – 0.860, p = 0.016). The most common cause of fasting interruption was hypoglycemia (67.4%), followed by hyperglycemia (24.8%). A weak positive correlation was observed between pre-Ramadan counseling scores and the number of completed fasting days (Spearman’s rho = 0.144; p = 0.025).

**Conclusion:**

Almost half of the participating Arab children, adolescents, and young adults with diabetes reported being permitted to fast during Ramadan. Older patients were more likely to be permitted to fast and completed more fasting days, and insulin pump users were more likely to be permitted to fast than those on multiple daily injections. A weak positive association was observed between counseling scores and the number of completed fasting days.

## 1 Introduction

During the holy month of Ramadan, Muslims abstain from food, drinks, and medications from dawn until sunset. Fasting becomes a religious obligation once a child reaches puberty. Depending on the latitude and season, daily fasting duration ranges between approximately 11 and 20 h (1, 2). For children and adolescents with diabetes mellitus (DM), these prolonged periods without caloric intake pose challenges in maintaining stable glycemic control and avoiding acute complications (3).

The global incidence of type 1 diabetes (T1D) is increasing at an estimated rate of 3–4% annually (4), and several Arab countries are ranked among the top nations worldwide for T1D incidence (5). Type 2 diabetes (T2D) is also observed in pediatric patients and is associated with increasing rates of obesity (6).

Adolescents with diabetes often experience difficulty achieving target blood glucose levels during fasting (1, 2). To address this issue, expert groups have proposed recommendations for managing diabetes during Ramadan, including patients on conventional insulin therapy, multiple daily injections (MDIs), and insulin pumps (3, 7, 8). However, most of these recommendations are based on expert opinions and small clinical series rather than large randomized controlled trials, emphasizing the need for individualized management and careful pre-Ramadan counseling.

A survey of pediatric endocrinologists in Arab countries revealed substantial variability in the management of children and adolescents during Ramadan (2). Continuous subcutaneous insulin infusion (CSII) may provide more stable glycemic profiles and allow more fasting days than MDIs (9, 10). Moreover, continuous glucose monitoring (CGM) is associated with a reduced risk of severe hypoglycemia and diabetic ketoacidosis (DKA) during Ramadan (11).

Fasting is associated with potential risks, including hypoglycemia, hyperglycemia, DKA, dehydration, and thrombosis, particularly in individuals with poor glycemic control, recent severe hypoglycemia or DKA, hypoglycemia unawareness, or limited access to medical care (1, 12, 13). Therefore, clinical guidelines recommend that patients considering fasting undergo a pre-Ramadan evaluation that addresses education, physical activity, glucose monitoring, meal planning, and insulin titration (14–16).

Despite being religiously exempt, many children and adolescents with diabetes choose to fast for all or part of Ramadan, driven by cultural, social, and personal motivations. This behavior has been documented in several studies (2, 3, 13, 14). However, there is limited systematic evidence regarding Ramadan fasting practices, safety, and pre-Ramadan counseling among children, adolescents, and young adults with diabetes living in Arab countries. Existing data are derived primarily from individual countries or small cohorts, and regional data remain limited.

Thus, the present study aimed to evaluate the experiences of Ramadan fasting among Arab children, adolescents, and youth with diabetes. Specifically, we examined the extent and content of pre-Ramadan counseling, frequency and safety of fasting, and factors associated with the ability to complete fasting days and fasting interruption. Most studies evaluating Ramadan fasting in people with diabetes have focused either on children and adolescents or on young adults as separate populations. We therefore included participants aged 7–25 years to provide a broader assessment of fasting practices across these age groups. Furthermore, individuals aged 18–25 years represent a transition-age period between adolescence and adulthood, during which diabetes self-management and fasting behaviors may differ from those observed in younger individuals.

## 2 Materials and methods

### 2.1 Study design and population

This cross-sectional questionnaire-based study included children, adolescents, and young adults with DM living in Arab countries. Eligible participants were aged 7–25 years, diagnosed with type 1 or type 2 diabetes, and had fasted or attempted to fast during Ramadan 2021. Parents completed the survey on behalf of younger children, whereas adolescents and youth responded themselves or with parental assistance. Because the survey was distributed through open electronic channels without a defined sampling frame, the response rate could not be calculated. No formal sample-size calculation was performed because this was an exploratory cross-sectional survey.

### 2.2 Questionnaire development and validation

An Arabic questionnaire was developed specifically for this study based on a review of the literature on Ramadan fasting and diabetes and previously published survey-based studies in pediatric diabetes populations. Content validity was evaluated by a panel consisting of two pediatric endocrinologists and one certified diabetes educator, who assessed the questionnaire for relevance, clarity, comprehensiveness, and clinical applicability. The preliminary version was pilot tested among 20 parents of children, adolescents and young adults with diabetes. Feedback obtained during pilot testing was used to refine wording and improve clarity. No formal psychometric reliability testing (e.g., internal consistency or test–retest reliability) was performed. The final questionnaire comprised three main sections:

1. Demographic and clinical data: Demographic and clinical information included age, sex, nationality, type and duration of diabetes, treatment regimen, glycated hemoglobin (HbA1c) level before Ramadan, use of a closed-loop insulin delivery system and continuous glucose monitoring (CGM), and the occurrence of diabetic ketoacidosis (DKA) or severe hypoglycemia during the 3 months preceding Ramadan.
2. Pre-Ramadan counseling: nine items evaluating whether the treating physician discussed key topics, including fasting planning, insulin dose modification, glucose monitoring, indications to break the fast, management of hypo- and hyperglycemia, hydration, healthy food choices, and exercise between iftar and suhoor. Each item was coded as “yes” (1) or “no” (0). The total counseling score (range, 0–9) was calculated by summing the affirmative responses.
3. Experience of Ramadan fasting: fasting desire (patient vs. patient and parent), physician permission to fast, number of completed fasting days, number of interrupted fasting days, DKA and severe hypoglycemia during Ramadan, and reasons for breaking the fast.

Episodes of DKA and severe hypoglycemia were self-reported by participants or their parents. Severe hypoglycemia was defined as a self-reported episode of seizure or loss of consciousness attributable to hypoglycemia. This simplified definition was intended to identify clinically significant events that could be readily recognized by participants and their families; therefore, less severe episodes requiring third-party assistance may not have been captured (15). For the purpose of this survey, DKA was defined as a self-reported episode requiring intensive care unit admission and intravenous insulin treatment. This simplified definition was intended to identify severe DKA events that could be readily recognized by participants and their families; therefore, milder episodes may not have been captured.

### 2.3 Data collection

The questionnaire was distributed electronically via social media platforms frequently used in Arab countries. Pediatric endocrinologists from the region were also invited to share the survey link with their patients and families. To minimize recall bias, data were collected within the first 10 days after the end of Ramadan. The participation was voluntary and anonymous. To minimize duplicate responses, participants were instructed to complete the questionnaire only once. Responses were reviewed for potential duplication based on response timestamps and similarities in demographic and clinical characteristics, and no apparent duplicate responses were identified. Thereafter, complete-case analysis was performed, and only participants with complete data for the variables included in each analysis were retained.

### 2.4 Ethical considerations

The current study was conducted in accordance with the principles of the Declaration of Helsinki. This study was reviewed and approved by the Regional Research Ethics Committee, Qassim University. Participants were first presented with information about the study. Electronic informed consent was obtained from participants aged ≥18 years or from parents/legal guardians of minors before access to the questionnaire. Completion of the anonymous online survey was considered evidence of voluntary participation, and participant assent was not separately documented.

### 2.5 Statistical analyses

Data were entered, cleaned, and analyzed using Microsoft Excel 2016 (Microsoft, Redmond, WA, USA) and SPSS version 26 (IBM Corp., Armonk, NY, USA). Categorical variables are summarized as frequencies and percentages. Continuous variables are summarized as means ± standard deviations (SDs) or medians (minimum–maximum), as appropriate.

The overall counseling score was treated as a continuous variable. The relationship between counseling score and the number of completed fasting days was assessed using Spearman’s rank correlation coefficient. Associations between physician permission to fast and participants’ sociodemographic and clinical characteristics were evaluated using the chi-squared test for categorical variables and the Mann–Whitney U test for continuous variables. Predictor reduction was performed by manual variable selection. Variables entered into the multivariable logistic regression model were selected based on clinical relevance, significant bivariate associations, and avoidance of temporal ambiguity. To reduce overfitting and ensure adequate statistical power, the number of predictors was minimized. The final adjusted model included only sex, HbA1c category, and CGM use as confounders. The events-per-variable (EPV) ratio for the final model exceeded the recommended threshold (EPV > 10), supporting the adequacy of the model.

Differences in the number of completed fasting days across sociodemographic and clinical characteristics were assessed using the Mann–Whitney U test or Kruskal–Wallis test, as appropriate. Data normality was evaluated using the Shapiro–Wilk test. All statistical tests were two-sided, and a p-value < 0.05 was considered statistically significant.

## 3 Results

### 3.1 Participant characteristics

In total, 242 patients with diabetes completed the questionnaire. The basic demographic and clinical characteristics are presented in **Table 1**. The largest age group was 13–17 years (50.8%), and slightly more than half of the participants were female (53.7%). Saudi nationals accounted for 70.2% of the respondents.

**Table 1:** Basic demographic profiles of the diabetic patients (n = 242).

Study variables	N (%)
Age group in years	
• 7–12 years	97 (40.1%)
• 13–17 years	123 (50.8%)
• 18–25 years	22 (09.1%)
Sex	
• Male	112 (46.3%)
• Female	130 (53.7%)
Nationality	
• Saudi	170 (70.2%)
• Oman	30 (12.4%)
• Egypt	15 (6.2%)
• Kuwait	13 (5.4%)
• Qatar	3 (1.2%)
• Iraq	3 (1.2%)
• UAE	3 (1.2%)
• Bahrain	2 (0.8%)
• Others	3 (1.2%)
Diabetes type	
• Type 1	232 (95.9%)
• Type 2	10 (04.1%)
Diagnosis of DM	
• < 1 year	54 (22.3%)
• 1–2 years	47 (19.4%)
• > 2–3 years	35 (14.5%)
• > 3 years	106 (43.8%)
DM regimen	
• Insulin pump	59 (24.4%)
• Insulin with Metformin	12 (5.0%)
• Multiple daily injections	171 (70.7%)
Continuous glucose monitoring (CGM)	
• Yes	180 (74.4%)
• No	62 (25.6%)

Values are presented as number (percentage) of participants; percentages are calculated from the total sample (n = 242) and may not sum to 100% because of rounding. No values in this table denote statistical significance, and the highlighting present in the proof is a residual revision marker that should be removed. DM, diabetes mellitus.

Most participants had type 1 diabetes (95.9%), while the remaining 4.1% had type 2 diabetes. Overall, 43.8% had diabetes for > 3 years. MDI was the most common treatment regimen (70.7%), followed by insulin pump therapy (24.4%). Only 5% were on insulin plus metformin. Approximately two-thirds of the cohort (66.9%) had uncontrolled HbA1c levels (≥ 7.5%) prior to Ramadan. A small proportion of patients (2.5%) used a closed-loop pump system, whereas 74.4% used CGM.

### 3.2 Pre-Ramadan counseling

The frequency of physician counseling regarding various aspects of fasting is shown in **Table 2**. Slightly more than half of the patients reported receiving counseling on planning fasting (56.6%), insulin dose adjustment (55.4%), and the importance of glucose monitoring during fasting (54.5%). Half of the participants were counseled about indications for breaking the fast (50.4%). Managing hypoglycemia (49.6%), maintaining adequate hydration (47.9%), managing hyperglycemia (47.1%), healthy food selection (38.0%), and exercising between the iftar and suhoor (36.4%) were less frequent.

**Table 2:** Physician counseling before Ramadan fasting (n = 242).

Readiness to fast Ramadan	Yes (%)
1. Discussion about planning the fasting	137 (56.6%)
2. Discussion about insulin dose modification, if necessary	134 (55.4%)
3. Discussion about the importance of monitoring glucose levels during fasting	132 (54.5%)
4. Discussion about the indication of fasting interruption	122 (50.4%)
5. Discussion about intervention for hypoglycemia during fasting	120 (49.6%)
6. Discussion about the importance of drinking plenty of fluids to avoid dehydration	116 (47.9%)
7. Discussion about intervention for hyperglycemia during fasting	114 (47.1%)
8. Discussion about healthy food selection	92 (38.0%)
9. Discussion about the importance of exercise between iftar and suhoor	88 (36.4%)
**Total score (mean ± SD)**	**4.35 ± 3.82**

Values are the number (percentage) of participants answering “yes” to each item. Each item was scored yes = 1 or no = 0, and the total counseling score was calculated by summing the affirmative responses (possible range 0–9). Bold type identifies the summary row (total counseling score) and does not indicate statistical significance. SD, standard deviation.

The mean counseling score was 4.35 ± 3.82 out of a possible 9 points, corresponding to approximately half of the maximum attainable score.

### 3.3 Readiness for fasting and fasting experience

**Table 3** summarizes readiness for Ramadan fasting and the fasting experience. In the 3 months preceding Ramadan, 11.5% of the participants experienced at least one episode of DKA and 5.0% had severe hypoglycemia. Hypoglycemia unawareness was reported by 26.0% of the respondents. The desire to fast originated from the patient alone in 74.4% of cases and from both patients and parents in 25.6%.

**Table 3:** Readiness to fast Ramadan and experience of fasting during Ramadan (n = 242).

Readiness to fast Ramadan	N (%)
DKA in the past 3 months	
• Yes	28 (11.5%)
• No	214 (88.4%)
Severe hypoglycemia in the past 3 months	
• Yes	12 (05.0%)
• No	230 (95.0%)
Affected with hypoglycemia unawareness	
• Yes	63 (26.0%)
• No	179 (74.0%)
Fasting desire	
• Patient	180 (74.4%)
• Patient and parent	62 (25.5%)
Visiting clinic in the past 4 months	
• Yes	203 (83.9%)
• No	39 (16.1%)
Did Physician allow fasting	
• Yes	110 (45.5%)
• No	132 (54.5%)

Experience of fasting during Ramadan	
DKA during Ramadan	
• Yes	15 (06.2%)
• No	227 (93.8%)
Episode of severe hypoglycemia during Ramadan	
• Yes	06 (02.5%)
• No	236 (97.5%)
	Median (min–max)
Number of completed fasting days	22.0 (0.0–30.0)
Number of interrupted fasting days	5.00 (0.0–30.0)

Values are presented as number (percentage) of participants, except for the final two rows, which are median (minimum–maximum). DKA, diabetic ketoacidosis.

Most participants (83.9%) had visited their diabetes clinic within the 4 months prior to Ramadan. Physicians permitted fasting in 45.5% of the patients.

During Ramadan, 15 (6.2%) participants experienced DKA and six (2.5%) participants experienced severe hypoglycemia. The median number of completed fasting days was 22 (range, 0–30), and the median number of interrupted fasting days was 5 (range, 0–30).

**Figure 1:**
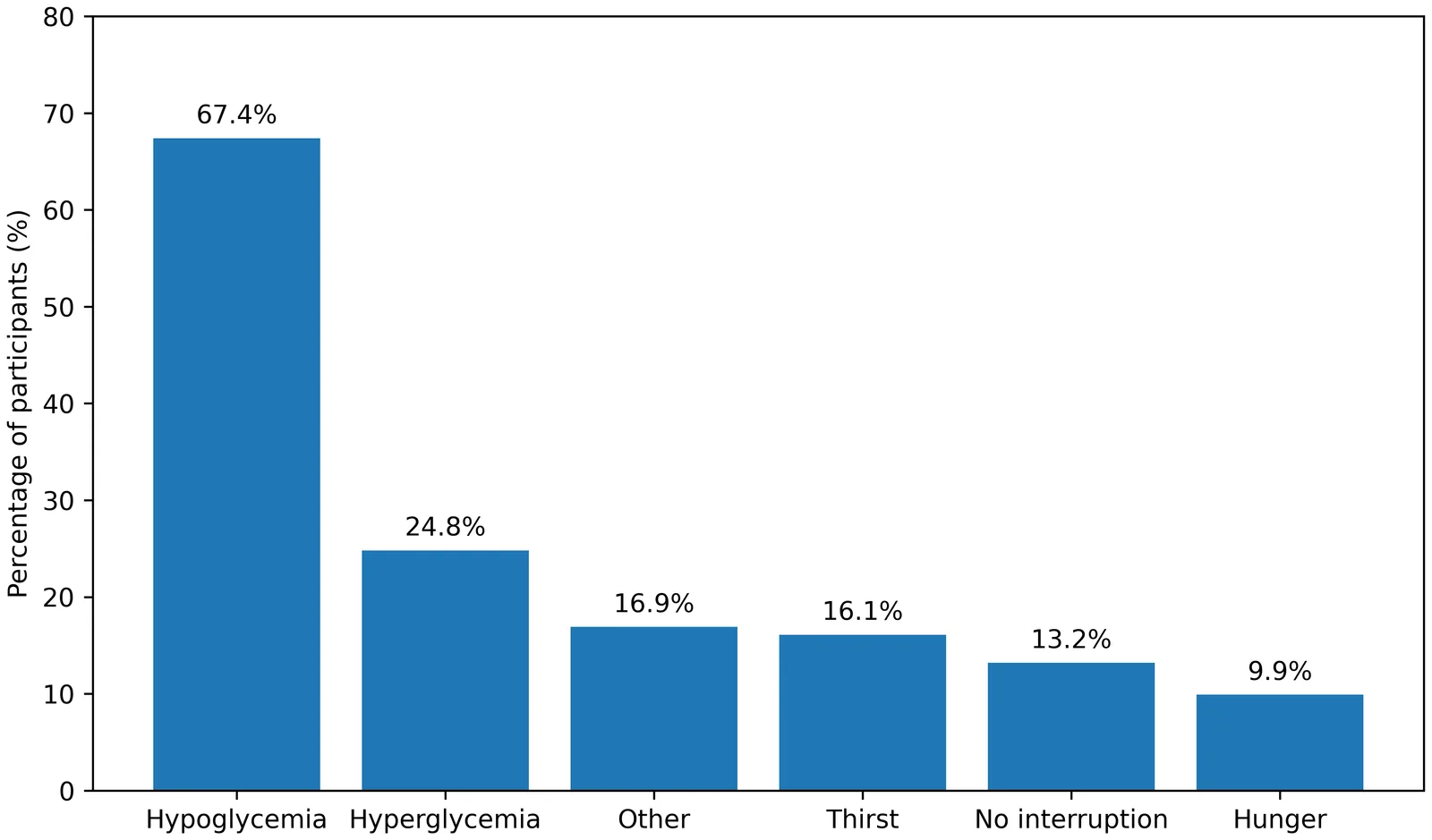
Cause of fasting interruption.

As shown in **Figure 1**, hypoglycemia was the most common cause of fasting interruption, followed by hyperglycemia, with thirst being the least frequent reason reported.

**Figure 2:**
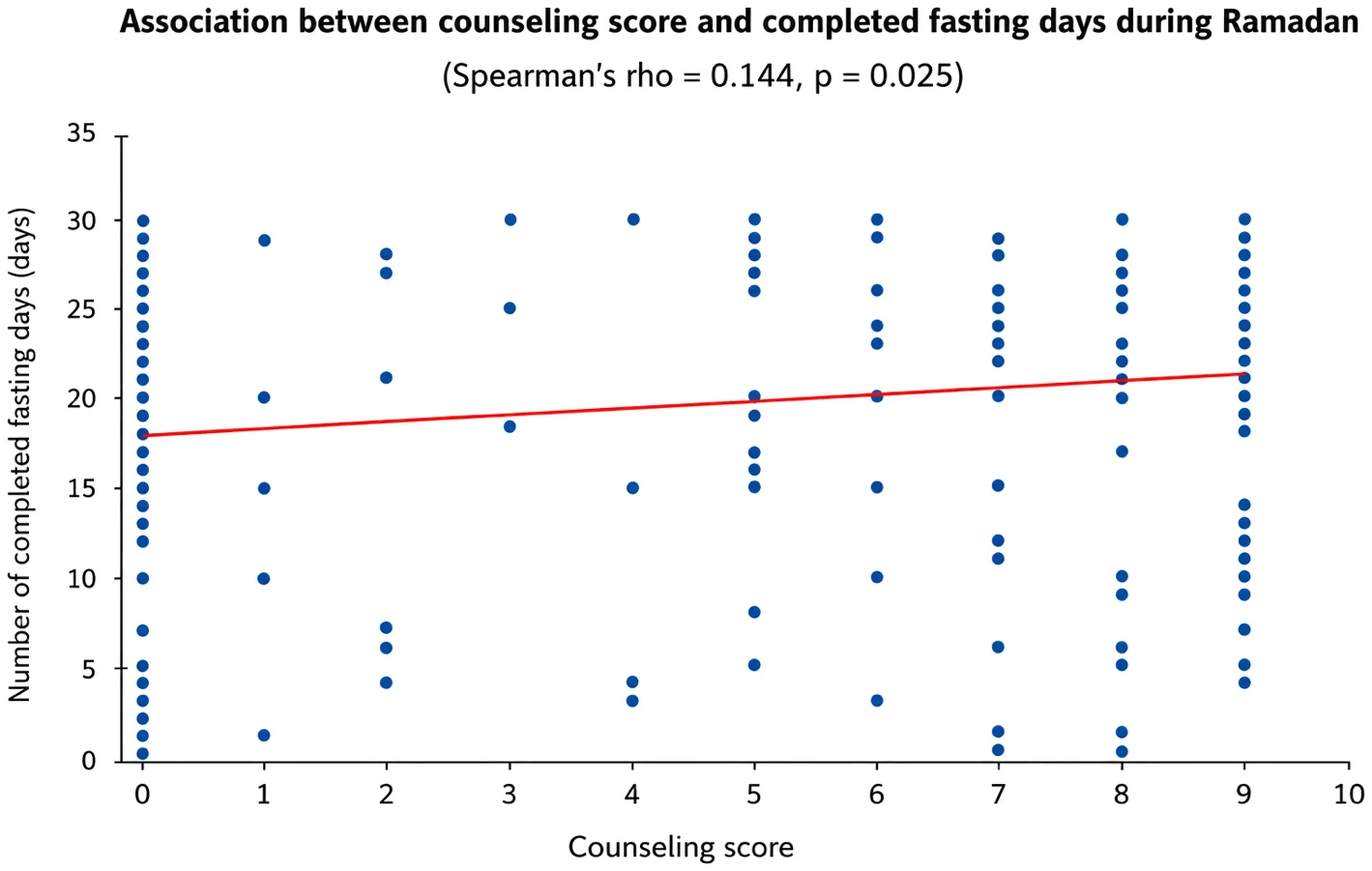
Correlation (Spearman correlation) between counseling score and the number of completed days in Ramadan fasting (n = 242).

A weak positive correlation was observed between the total counseling score and the number of completed fasting days (Spearman’s rho = 0.144, p = 0.025; **Figure 2**).

### 3.4 Factors associated with permission to fast

The relationship between physicians’ permission to fast and sociodemographic and clinical variables is shown in **Table 4**. Older participants (aged 13–25 years) were more likely to be allowed to fast compared with younger children (aged 7–12 years) (p = 0.033). No significant association was found between permission to fast and sex, diabetes type, diabetes duration, HbA1c level, or CGM use.

**Table 4:** Relationship between allowing to fast during Ramadan and sociodemographic data of patients with diabetes ^(n=242)^.

Factor	Allowed to fast		P-value ^§^
	No N (%) ^(n=132)^	Yes N (%) ^(n=110)^	
Age group in years			
• 7–12 years	61 (46.2%)	36 (32.7%)	**0.033** ^**^
• 13–25 years	71 (53.8%)	74 (67.3%)	
Sex			
• Male	66 (50.0%)	46 (41.8%)	0.204
• Female	66 (50.0%)	64 (58.2%)	
Diabetes type			
• Type 1	128 (97.0%)	104 (94.5%)	0.345
• Type 2	04 (03.0%)	06 (05.5%)	
Diagnosis of DM			
• < 1 year	37 (28.0%)	17 (15.5%)	0.083
• 1–2 years	27 (20.5%)	20 (18.2%)	
• > 2–3 years	17 (12.9%)	18 (16.4%)	
• > 3 years	51 (38.6%)	55 (50.0%)	
DM regimen			
• Insulin pump	24 (18.2%)	35 (31.8%)	**0.006** ^**^
• Multiple daily injections	103 (78.0%)	68 (61.8%)	
• Insulin and metformin	05 (03.8%)	07 (06.4%)	
HbA1c level before Ramadan			
• Controlled (< 7.5%)	40 (30.3%)	40 (36.4%)	0.318
• Uncontrolled (≥ 7.5%)	92 (69.7%)	70 (63.6%)	
CGM			
• Yes	100 (75.8%)	80 (72.7%)	0.591
• No	32 (24.2%)	30 (27.3%)	
Number of completed fasting days	20.0 (0.00–30.0)	24.0 (1.00–30.0)	**0.002** ^**^
Number of interrupted fasting days	5.00 (0.00–30.0)	4.00 (0.00–26.0)	0.152

§ P-value was calculated using the chi-squared test.

‡ P-value was calculated using the Mann–Whitney U test.

** Significant at p < 0.05 level.

Values are number (percentage) of participants, except for the final two rows, which are median (minimum–maximum). Bold type indicates statistical significance.DM, diabetes mellitus; CGM, continuous glucose monitoring.

Regarding treatment regimen, both insulin pump users and those receiving insulin plus metformin were allowed to fast more frequently than patients receiving MDI alone (p = 0.006). Participants who were permitted to fast had a higher median number of fasting days than those who were not permitted to fast (24 vs. 20 days, p = 0.002).

In the adjusted multivariable logistic regression model, age and diabetes treatment regimen emerged as significant independent predictors of physician permission to fast during Ramadan. Participants aged 13–25 years had nearly twice the odds of receiving physician permission to fast compared with those aged 7–12 years (AOR = 1.951, 95% CI: 1.132–3.363, p = 0.016). In addition, patients receiving multiple daily injections had significantly lower odds of being permitted to fast than those using insulin pump therapy (AOR = 0.444, 95% CI: 0.230–0.860, p = 0.016). These associations remained significant after adjustment for sex, HbA1c category, and CGM use, indicating that age and treatment regimen were the principal factors associated with physicians’ fasting recommendations (**Table 5**).

**Table 5:** Multivariate regression analysis to determine the significant independent predictor of allowing fasting during Ramadan among patients with diabetes ^(n=242)^.

Factor	AOR	95% CI	P-value
Age group in years			
• 7–12 years	Ref		
• 13–25 years	1.951	1.132 – 3.363	0.016 **
DM regimen ^†^			
• Insulin pump	Ref		
• Multiple daily injections	0.444	0.230 – 0.860	0.016 **

Adjusted for sex, HbA1c, and CGM.

† Insulin + Metformin category for DM regimen was excluded from the adjusted model because of small subgroup size (N = 12).

AOR, adjusted odds ratio; CI, confidence interval; DM, diabetes mellitus; CGM, continuous glucose monitoring; Ref, reference category.

**Significant at p <0.05 level.

Bold type indicates variables that reached statistical significance (p < 0.05) in the adjusted model; the highlighting present in the proof carries no additional meaning and should be removed.

### 3.5 Factors associated with number of completed fasting days

**Table 6** shows the association between sociodemographic and clinical variables and the number of fasting days completed. Older age (aged 13–25 years) was associated with a higher median number of completed days than younger age (p < 0.001). Patients with T2D had a significantly higher median number of fasting days than those with T1D (27.5 vs. 21 days, p = 0.004).

**Table 6:** Association between the number of completed fasting days and sociodemographic data of the diabetic patients (n = 242).

Factor	Number of completed days fast Median (min–max)	P-value
Age group in years		
• 7–12 years	20.0 (0.0–30.0)	< 0.001^**,^ ^a^
• 13–25 years	24.0 (0.0–30.0)	
Sex		
• Male	25.0 (0.0–30.0)	0.056^a^
• Female	20.0 (0.0–30.0)	
DM type		
• Type 1	21.0 (0.0–30.0)	**0.004** ^**^ ^a^
• Type 2	27.5 (20.0–30.0)	
Diagnosis of DM		
• < 1 year	20.0 (1.0–30.0)	0.587^b^
• 1–2 years	23.0 (0.0–30.0)	
• > 2–3 years	20.0 (0.0–30.0)	
• > 3 years	23.0 (0.0–30.0)	
DM regimen		
• Insulin pump	22.0 (0.0–30.0)	0.044^**,^ ^b^
• Multiple daily injections	21.0 (0.0–30.0)	
• Insulin and metformin	27.5 (20.0–30.0)	
HbA1c level before Ramadan		
• Controlled (< 7.5%)	20.0 (0.0–30.0)	0.092^a^
• Uncontrolled (≥ 7.5%)	23.0 (0.0–30.0)	
DKA during Ramadan		
• Yes	23.0 (5.0–30.0)	0.730^a^
• No	22.0 (0.0–30.0)	
Severe hypoglycemia during Ramadan		
• Yes	23.5 (12.0–30.0)	0.540^a^
• No	21.5 (0.0–30.0)	

a P-value was calculated using the Mann–Whitney U test.

b P-value was calculated using the Kruskal–Wallis test.

** Significant at p < 0.05 level.

Bold type indicates statistical significance.

DM, diabetes mellitus; DKA, diabetic ketoacidosis.

The treatment regimen also influenced the fasting duration; patients treated with insulin plus metformin had a higher median number of completed fasting days than those treated with an insulin pump or MDI (p = 0.044). There were no significant associations between the completed fasting days and sex, diabetes duration, HbA1c level, or the occurrence of DKA or severe hypoglycemia during Ramadan.

## 4 Discussion

This multicenter survey provides insights into the real-world experiences of Ramadan fasting among Arab children, adolescents, and youth with diabetes. Almost half of the participants were permitted to fast by their physicians, and many of these patients completed a substantial number of fasting days. Overall, severe hypoglycemia was uncommon during Ramadan; however, DKA was reported by a clinically important minority of participants, indicating that fasting was not without risk. Historically, patients with T1D were often advised not to fast because of concerns regarding hypoglycemia and DKA (13, 14). More recent ISPAD and IDF–DAR guidelines, together with accumulating clinical experience, suggest that, with appropriate patient selection, individualized risk stratification, and structured pre-Ramadan education, some children and adolescents with diabetes can fast safely (1, 5, 6, 16–18). The present study supports this view, showing that a carefully selected subgroup of youth—with physician approval and pre-Ramadan counseling—was able to fast with an acceptable rate of complications.

The proportion of patients allowed to fast in this study (45.5%) was lower than the percentage of pediatricians in the United Arab Emirates (UAE) who reported that they would permit fasting for their patients (79.6%) (13). This discrepancy may reflect differences in study populations, sampling methods, age distribution, access to diabetes technology and healthcare resources, outcome definitions, and clinician attitudes toward Ramadan fasting (2).

Our findings indicate that older age is a key factor influencing both permission to fast and fasting success. Although studies from Kuwait and the UAE did not identify age as a significant determinant (10, 13, 18), a study from Bangladesh reported that older age was associated with better fasting outcomes, possibly reflecting greater self-management skills and lifestyle stability among adolescents and young adults compared with younger children (3).

We also observed that patients with T2D and those treated with insulin combined with metformin had a greater number of fasting days than those with T1D. This may be explained by the relatively preserved endogenous insulin secretion and lower glycemic variability, which reduce the risk of severe hypo- or hyperglycemia. Similar findings were reported in the EPIDIAR study, which demonstrated that individuals with T2D experienced fewer severe hypoglycemic events and were more likely to complete fasting during Ramadan than those with T1D. Overall, these findings support the notion that diabetes type and treatment regimen play a significant role in fasting tolerance, including that in adolescent populations (19, 20). Although participants with type 2 diabetes appeared more likely to complete Ramadan fasting, this finding should be interpreted with caution because only a small number of participants had type 2 diabetes, limiting the precision and generalizability of subgroup comparisons.

Most participants were managed with MDI; however, insulin pump users were significantly more likely to fast. CSII may facilitate more flexible insulin dose adjustments and reduce glucose variability during Ramadan (1, 10). Nevertheless, in our cohort, MDI users also completed a considerable number of fasting days, highlighting that safe fasting is possible with different regimens if patients are properly educated. The relatively high use of CGM (74.4%) in our cohort may also have influenced the observed fasting experiences and complication rates. Participants recruited through pediatric endocrinology networks and social media platforms may represent a more engaged and better-resourced population with greater access to diabetes technology, specialist care, and pre-Ramadan counseling. Consequently, the fasting outcomes reported in this study may not be fully generalizable to lower-resource populations or individuals with more limited access to diabetes care.

Hypoglycemia was the most common reason for breaking the fast, which is consistent with previous reports from Bangladesh, the UAE, and Saudi Arabia (3, 10, 11). This emphasizes the importance of educating patients about adjusting insulin doses, frequent glucose monitoring, recognizing the symptoms of hypoglycemia, and the necessity of terminating fasting when hypoglycemia occurs (1, 2).

We observed a slight downward trend in the incidence of DKA and severe hypoglycemia during Ramadan compared with that 3 months prior to Ramadan. The incidence rate of DKA decreased from 11.5% before Ramadan to 6.2% during the fasting period, whereas that of severe hypoglycemia decreased from 5.0% to 2.5%. Although the frequency of DKA and severe hypoglycemia reported during Ramadan appeared lower than that reported in the preceding 3 months, the differing observation periods preclude direct comparison of event rates. Therefore, these findings should be interpreted descriptively rather than as evidence of a reduction in complications during Ramadan. These findings are consistent with a cross-sectional study involving 20 children that showed that patients with better glycemic control (HbA1c ≤ 8.5%) were able to fast for more days and experienced significantly fewer hypoglycemic episodes than those with poorer glycemic control (HbA1c > 8.5%) (18). Similarly, Deeb et al. reported that among patients who fasted for more than half of Ramadan or throughout the entire month, over half experienced at least one episode of hypoglycemia and nearly one-third reported hyperglycemia, with only one documented case of DKA (21). In contrast, Musleh et al. and Al-Agha et al. reported no episodes of severe hypoglycemia or DKA during Ramadan fasting, demonstrating more favorable outcomes, potentially attributable to differences in patient selection, education, and clinical supervision (10, 11). Although many participants were able to complete Ramadan fasting, the occurrence of DKA in 6.2% of participants highlights that fasting is not without risk. These findings underscore the importance of individualized risk assessment, appropriate patient selection, and structured pre-Ramadan counseling to minimize the risk of acute metabolic complications.

A statistically significant but weak positive association was observed between counseling scores and the number of completed fasting days (Spearman’s rho = 0.144). Although this finding suggests a possible relationship between counseling and fasting behavior, the effect size was small and its clinical significance is uncertain. Furthermore, causality cannot be inferred because of the cross-sectional study design. Although causality cannot be inferred from this cross-sectional study, this finding is consistent with current recommendations emphasizing structured pre-Ramadan education for individuals with diabetes who choose to fast (1, 2, 12, 14, 21).

Future studies should explore additional approaches to individualized risk assessment before Ramadan fasting, including whether the quality and comprehensiveness of pre-Ramadan counseling are associated with a reduced risk of fasting-related complications. Emerging cardiovascular imaging modalities, including speckle-tracking echocardiography, have shown promise for detecting subclinical myocardial dysfunction in young individuals with metabolic disease, although their role in guiding fasting recommendations remains unknown (22).

### 4.1 Strengths and limitations

This study has several strengths, including its focus on a relatively large cohort of Arab children and youth with diabetes across multiple countries and its assessment of both pre-Ramadan counseling and fasting outcomes.

However, several limitations should be considered. The cross-sectional design precludes causal inference, and the reliance on self-reported data may have introduced recall and reporting bias. HbA1c levels, glucose-monitoring data, and acute complications were not independently verified through medical records. The survey was distributed through social media using a convenience sampling approach, which may have introduced selection bias and limited generalizability. In addition, most participants were from Saudi Arabia, the response rate could not be determined, and the small number of participants with type 2 diabetes limits subgroup-specific conclusions. Furthermore, the limited number of diabetic ketoacidosis and severe hypoglycemia events precluded analyses to identify independent predictors or meaningful subgroup comparisons according to age. Larger prospective studies are needed to identify factors associated with fasting-related adverse events across different age groups. Finally, although the questionnaire underwent expert review and pilot testing, formal psychometric reliability testing was not performed.

## 5 Conclusion

In this online cross-sectional sample of Arab children, adolescents, and young adults with diabetes, many participants reported completing Ramadan fasting days and receiving pre-Ramadan counseling. However, acute complications, including diabetic ketoacidosis and severe hypoglycemia, were reported, highlighting that fasting is not without risk. These findings should be interpreted with caution because outcomes were self-reported and participants were recruited through a non-probabilistic convenience sampling approach.

This study supports current recommendations regarding structured pre-Ramadan counseling for young people with diabetes who choose to fast. Future prospective studies with objective metabolic monitoring are required to further refine the risk stratification and fasting protocols in this population.

## Data Availability

The original contributions presented in the study are included in the article/**Supplementary Material**. Further inquiries can be directed to the corresponding author.
